# Dr. Roane

**Published:** 1833

**Authors:** 


### XI. Medical Obituary.

James Roane, M. D. of *Nashville, Tenn.* This gentleman died of Epidemic Cholera, on the 27th of February last, at the age of 43. He was a native of that state, in which, for many years, he sustained a high rank as a physician and a gentleman. At the time of his death, he was President of the Medical Society for regulating the practice of physic, in Tennessee. In our cotemporary of Lexington, we observe a notice of his life and character, from its accomplished Editor, Professor Yandell who knew him personally, from which we extract the following honorable and merited tribute.—'The qualities to which he owed his enviable popularity, may be summed up in these words—his industry made him learned; his punctuality in business secured him the confidence of the public; his liberality won for him the admiration and respect of his professional rivals; and the kindness of his manners, and the gentleness of his temper rendered him the idol of his patients." The death of such a physiciaa, in the meridian of his usefulness, is a public calamity.

